# A proposal for a dipole-generated BLUF domain mechanism

**DOI:** 10.3389/fmolb.2015.00062

**Published:** 2015-11-03

**Authors:** Tilo Mathes, Jan P. Götze

**Affiliations:** ^1^Biophysics Group, Department of Physics and Astronomy, Faculty of Sciences, Vrije UniversiteitAmsterdam, Netherlands; ^2^Institut für Biologie/Experimentelle Biophysik, Humboldt Universität zu BerlinBerlin, Germany; ^3^School of Chemistry, University of St AndrewsSt Andrews, UK

**Keywords:** BLUF, flavin, signal transduction, protein structure, electron transfer

## Abstract

The resting and signaling structures of the blue-light sensing using flavin (BLUF) photoreceptor domains are still controversially debated due to differences in the molecular models obtained by crystal and NMR structures. Photocycles for the given preferred structural framework have been established, but a unifying picture combining experiment and theory remains elusive. We summarize present work on the AppA BLUF domain from both experiment and theory. We focus on IR and UV/vis spectra, and to what extent theory was able to reproduce experimental data and predict the structural changes upon formation of the signaling state. We find that the experimental observables can be theoretically reproduced employing any structural model, as long as the orientation of the signaling essential Gln63 and its tautomer state are a choice of the modeler. We also observe that few approaches are comparative, e.g., by considering all structures in the same context. Based on recent experimental findings and a few basic calculations, we suggest the possibility for a BLUF activation mechanism that only relies on electron transfer and its effect on the local electrostatics, not requiring an associated proton transfer. In this regard, we investigate the impact of dispersion correction on the interaction energies arising from weakly bound amino acids.

## BLUF photoreceptors

### Flavins as chromophores in biological photoreceptors

Most biological organisms depend on the ability to evaluate ambient light levels to face environmental challenges and to adjust their behavior and metabolism appropriately. These sensory mechanisms are especially crucial for plants or photosynthetically active bacteria. Various molecular solutions and mechanisms have been described over the years, which allow for a reaction to different light levels and qualities. A group of blue light receptor proteins employ flavin chromophores and are classified as cryptochromes (cry), light-oxygen-voltage (LOV), and blue light sensing using FAD (BLUF) photoreceptors. These proteins regulate gene expression and enzyme activities via a ubiquitously available cofactor, thus being interesting targets for the field of optogenetics; for corresponding reviews (see Losi, 2007; Hegemann, 2008; Möglich et al., 2010; Losi and Gärtner, 2012). The photoreceptor domains form a sheath around the flavin photosensor and translate light into biological information upon excitation of the chromophore. The flavin molecules used in these photoreceptors are riboflavin (RF, vitamin B_2_) derived cofactors namely flavin-mononucleotide (FMN) and flavin-adenine-dinucleotide (FAD). A simpler flavin is lumiflavin (LF), which is the model compound primarily used in computational studies. The photochemistry of all these compounds is almost exclusively determined by the isoalloxazine moiety and widely considered to be identical in BLUF domains (Laan et al., 2004).

As the employed chromophore is identical, any difference in photochemistry between the flavin-based photoreceptors (cry, LOV, and BLUF) must arise from the protein environment. Hence, the dynamic nature of chromophore-protein interaction is a matter of utmost interest, as insight into this relationship would allow scientists to rationally design and control sensitivity, quantum yield, and receptor-target interaction for optogenetic application and synthetic biology. As this information is sometimes problematic to obtain experimentally, computational methods provide very attractive approaches with molecular resolution to investigate the corresponding parts/modules of the given protein.

**Figure 1:**
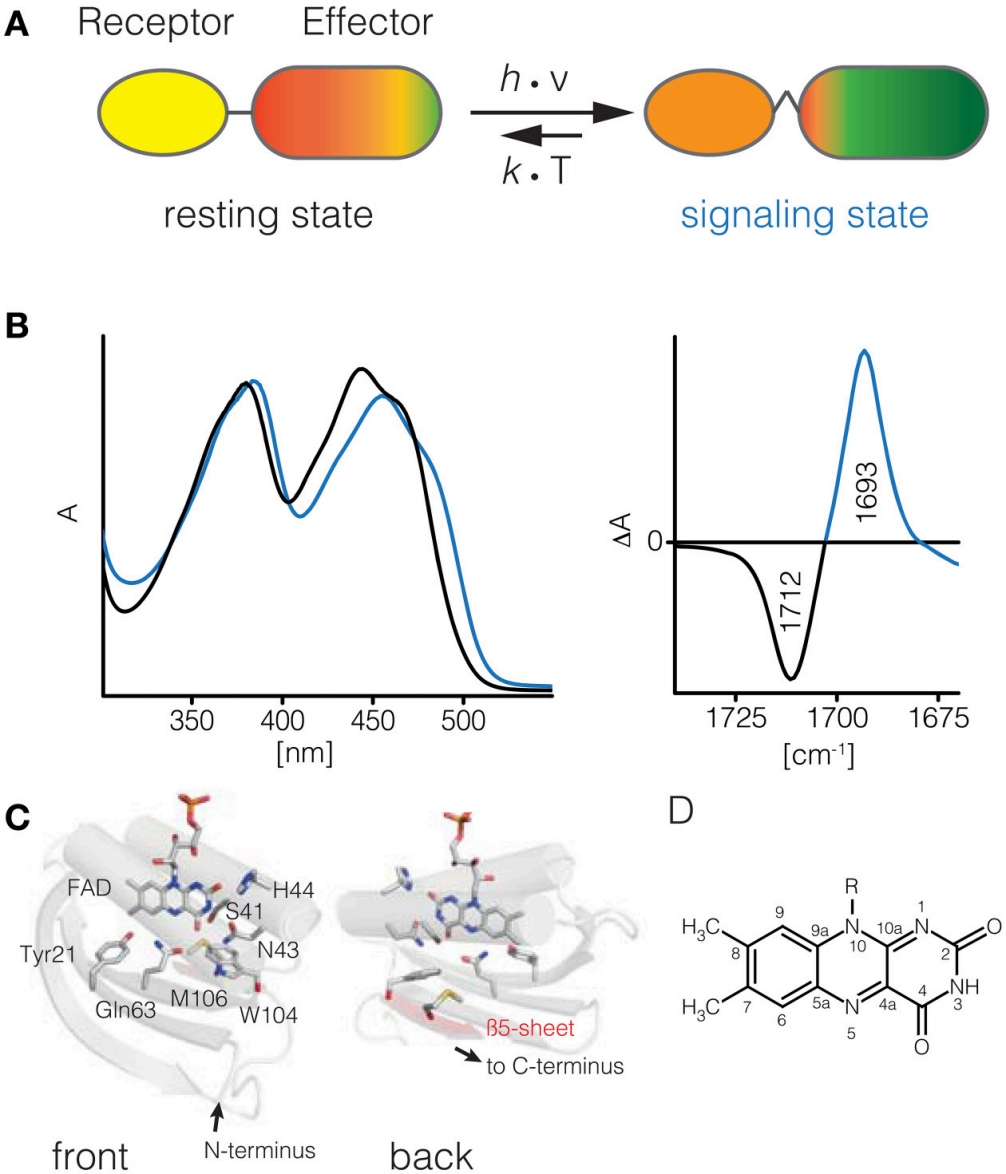
**BLUF photoreceptors**. Upon illumination of the BLUF domain (receptor) structural changes are transmitted to its cognate effector domain and induce an increased activity **(A)**. These structural changes thermally relax into the resting state. The UV/vis spectrum of BLUF domains shifts by about 10–15 nm to red upon illumination **(B)**. In the signaling-minus-resting state FTIR difference spectrum a characteristic downshift of the C4 = O carbonyl signature is observed **(C)**. Structural model of the AppA BLUF domain and topology map (**D**, PDB ID 1YRX). Isoalloxazine moiety of flavin cofactors **(E)**.

The general principle of a protein photoreceptor domain is rather simple: The chromophore/protein complex must be able to switch upon excitation of the chromophore from an equilibrium conformation, the resting state^1^, to a metastable signaling state that is present for a certain amount of time before relaxing back to the resting state (Figure 1A). Depending on the photoreceptor domain at hand, the conformational change is facilitated by a different photochemical reaction. A common concept is light induced electron transfer (ET) from the protein environment to the flavin chromophore itself. Excited flavins either in the singlet or even more so in the triplet excited state represent efficient electron acceptors (Porcal et al., 2003) that receive electrons from aromatic amino acids like tyrosine and tryptophan (cry and BLUF; Gauden et al., 2007; Song et al., 2007) or the sulfur atom of cysteine side chains (LOV; Holzer et al., 2002). The photocycles of the different receptor domains diverge after this initial electron transfer: In cry radical states are stabilized; in BLUF domains, the hydrogen bond network is reordered without any chemical change of the flavin chromophore; and LOV domains form a covalent bond to the protein environment through a cysteinyl-flavin adduct. For more details, we point to Möglich et al. (2010) and references therein.

### Photoswitching in bluf domains

Here, we will focus on the modular blue light sensing using FAD (BLUF) photoreceptors. Their function has been first described in 2002 (Gomelsky and Klug, 2002; Iseki et al., 2002), and substantial progress has been made since then to elucidate the molecular events following flavin excitation and subsequent communication to the effector domain/protein. BLUF domains require a single flavin excitation event to form the signaling state conformation that has a half-life from few seconds up to tens of minutes. This signaling state conformation is represented by a subtle rearrangement of the hydrogen bonding network around the flavin. The redox state of the flavin in the signaling state remains identical to the resting state, making the BLUF domain possibly the chemically simplest and probably most intriguing blue light receptor domain. The signaling state differs from the resting state in several well-documented aspects consistent throughout the so far investigated BLUF domains (Kennis and Mathes, 2013): The UV/vis spectrum of the signaling state is shifted by 0.1–0.15 eV to lower energies, corresponding to a shift of 10–15 nm of the first bright π → π^*^ transition to longer wavelengths (Figure 1B). The most prominent feature of the IR spectrum is the downshift of the FMN(C4 = O4) stretch peak by about 20 cm^−1^ in the signaling state, indicating a strong change in hydrogen bonding to this particular functional group (Figures 1C,E). These observations are consistent throughout all investigated BLUF domains and have as such been used as a primary quality criterion for computational studies (see Computational Studies on BLUF). To accomplish this photochromic shift two conserved residues, Tyr21 and Gln63, are required.

### Molecular models of bluf domains

For BLUF domains several molecular models based on X-ray crystallographic and NMR spectroscopic data have been available since 2005 (Anderson et al., 2005; Jung et al., 2005, 2006; Kita et al., 2005; Grinstead et al., 2006; Yuan et al., 2006; Barends et al., 2009; Wu and Gardner, 2009; Winkler et al., 2013). Yet, for BLUF domains, the structural data sparked significant controversy as molecular models of even the same BLUF protein provided ambiguous geometries. The BLUF architecture comprises roughly 100–110 amino acids in size, up to 140 if the C-terminal capping α-helices are included. The common secondary structure motif is a Ferredoxin-like βαββαββ fold. The β-motifs form an anti-parallel β-sheet, which together with the two core α-helices encapsules the FMN chromophore via non-covalent interactions (see Figure 1D).

In the so far available crystal structures of the AppA BLUF domain significant differences have been observed for the conformation of the Trp104 residue, which was reported to be located either close/proximal (Trp-in; Anderson et al., 2005) to the FMN or located on the surface/distal to the chromophore (Trp-out; Jung et al., 2006) of the BLUF domains. The Trp-in conformation is found for the WT BLUF domain while the Trp-out conformation is present in an N-terminal mutant in the presence of high concentrations of β-mercaptoethanol and in the full-length protein (Winkler et al., 2013). Accordingly, the Trp side chain conformation is either in the range for formation of a N_ε_-H hydrogen bond to Gln63(O), or it is found on the surface of the β-sheet, partially exposed to the surface. Note that “surface exposition” is not identical to “solvent exposition,” the β-sheet is known to be either a dimerization interface (Anderson et al., 2005; Jung et al., 2005, 2006; Wu and Gardner, 2009) or protected from the solvent by the C-terminal capping helices (Kita et al., 2005; Yuan et al., 2006; Barends et al., 2009). Other BLUF domains like BlrB, TePixD, and SyPixD, which are present as dimeric (BlrB) or decameric (PixD) complexes in the crystal and solution show predominantly the Trp-out conformation. Despite the wide variety of available structures, most theoretical studies focus on the AppA BLUF domain as will be discussed below.

A more subtle yet crucial ambiguity is the side-chain conformation of Gln63. The resolution of the currently available crystal structures is not sufficient to distinguish between the amidic oxygen and nitrogen. Therefore, different conformations based on the proposed hydrogen bond pattern from Gln63 to Trp104 and from Tyr21 to Gln63 were proposed.

So far, no crystal or NMR structure prepared under light-adapted conditions has been reported. However, it is still conceivable that some of the reported dark-adapted structures contain features of the light-adapted state due to crystal packing, conformational selection by crystallization or photoactivation by synchrotron radiation. Some crystals were investigated by micro-UV-vis-spectrophotometry to demonstrate photoswitching functionality and dark state conformation (Jung et al., 2006). However, as the photochromic switch may be decoupled from the signal transduction (see discussion on signal transduction below), the UV/vis spectral properties may provide a misleading picture. Additionally, crystal structures recorded at cryogenic temperature may exhibit different side chain conformations than under RT conditions relevant for most spectroscopic investigations (Fraser et al., 2011).

In addition to studies involving the full protein, tryptophan, and tyrosine residues have been probed selectively by steady state NMR and UV resonance Raman spectroscopy corroborating the Trp-in position for the resting state in the truncated AppA BLUF domain (Grinstead et al., 2006; Unno et al., 2006, 2010). NMR spectroscopy, however, also showed that the region harboring Trp104 and Met106 is very flexible and generally influenced by the hydrogen bond network around the flavin (Grinstead et al., 2006; Wu et al., 2008; Yuan et al., 2011). Another commonly used approach for probing the conformation and environment of tryptophan residues in proteins is tryptophan fluorescence spectroscopy (Reshetnyak et al., 2001). These investigations showed that the environment of Trp104 depends on the length of the C-terminal truncation in AppA-BLUF constructs but does not change strongly upon formation of the signaling state and clearly does not become fully solvent exposed as suggested by some crystal structures (Toh et al., 2008; Dragnea et al., 2009). These studies, however, differ in their conclusions whether Trp104 is located in proximal Trp-in (Toh et al., 2008) or distal Trp-out location to the flavin (Dragnea et al., 2009). Similarly, no significant change in the tryptophan environment was observed for SyPixD (Yuan et al., 2011).

Selective isotope labeling of the tyrosine side chain in AppA and TePixD provided indirect information on the orientation of the hydrogen bonded glutamine residue. The presence of an unusually strong hydrogen bond donated from Tyr-21 in the signaling state of AppA was interpreted as incompatible with hydrogen bonding to the glutamine side chain amidic nitrogen (Iwata et al., 2011), while a previous study on TePixD concluded that both dark and light state properties of tyrosine are compatible with H-bonding to oxygen but could not fully exclude alternative orientations (Takahashi et al., 2007). A study on SyPixD with a different tyrosine isotope labeling pattern gave similar results and clearly showed that the structural changes are confined to the phenolic hydroxyl group without any changes to the aromatic nature of the phenyl ring (Mehlhorn et al., 2013).

### Signal transduction in bluf domains

On top of these apparent controversies on the tryptophan/methionine orientation also its relevance of for signal transduction remains to be considered. Biochemical and physiological experiments that investigate signal transduction of BLUF photoreceptors by using site-directed mutations provided inconsistent effects within the BLUF protein family. In AppA, the removal of Trp104 or Met106 each disrupt the signaling process *in vivo*, while the light-induced formation of the red shifted signaling state is not affected (Masuda et al., 2007). Similar results have been obtained for the bacterial photoactivated adenlylyl cyclase (bPAC), where signaling and photochemistry are also clearly decoupled by the corresponding mutations (Stierl et al., 2014). However, in other BLUF domains that also contain the semi-conserved tryptophan only the conserved Met106 is crucial for signaling (Masuda et al., 2008). Replacement of Trp104, however, has a significant effect on the structural dynamics and stability of the light-adapted state. In AppA, the Trp104Ala or Trp104Phe mutation reduces the stability of the signaling state, which is also witnessed by the absence of a secondary structure change involving the β5 sheet in light-minus-dark FTIR difference spectra (Masuda et al., 2005a; Majerus et al., 2007). Confusingly, the analogous mutations in SyPixD and bPAC lead to stabilization, by exchange to Phe, or destabilization, by exchange to Ala, of the red shifted signaling state (Mathes, 2007; Bonetti et al., 2009; Stierl et al., 2014).

In this regard it should also be noted that even some variants lacking essential residues like the conserved glutamine or tyrosine still show light-induced signaling, however to significantly smaller extent (Metz et al., 2010; Stierl et al., 2014). These observations suggest that already the formation of radical intermediates in BLUF domains may be sufficient to drive signal transduction. Similar implications have been recently obtained by time resolved spectroscopic studies (Fujisawa et al., 2014; Fudim et al., 2015).

### Photodynamics of bluf domains

Although steady state data are usually very robust and informative, they may be of very limited use for the elucidation of reaction mechanisms. The ultrafast hydrogen-bond rearrangement in BLUF domains in less than 1 ns (as indicated by the red shifted absorbance of the flavin) requires pump-probe spectroscopy and is limited to optical methods. A particular feature of the BLUF photodynamics is a highly multi-exponential excited state decay behavior (Dragnea et al., 2005; Gauden et al., 2005, 2006; Bonetti et al., 2008, 2009; Shibata et al., 2009; Mathes et al., 2011, 2012a), indicating a structural microheterogeneity in the resting state, which is most likely due to minor variations of the mutual flavin/tyrosine orientation. Depending on the BLUF domain at hand the observable excited state lifetimes range from few ps up to several ns and severely limit experimental access to reaction intermediates that are formed with faster (inverted) kinetics. So far in only two BLUF proteins, SyPixD and PapB, reaction intermediates have been identified (Gauden et al., 2006; Fujisawa et al., 2014). Both proteins show the formation of a neutral flavin semiquinone radical that decays into the red-shifted signaling state. For SyPixD also an anionic semiquinone radical intermediate preceding the formation of the neutral form has been observed that illustrates a strictly sequential electron transfer (ET) proton transfer (PT) process. In addition, a signature for the corresponding electron donor (Tyr21) has been obtained by transient IR spectroscopy (Bonetti et al., 2008). The significance for these intermediates for signaling state generation has been challenged recently (Lukacs et al., 2014) but is strongly supported by site-directed mutagenesis and redox tuning of the tyrosine electron donor, that directly affects the rate of electron transfer and the quantum yield of signaling state formation (Mathes et al., 2012a). Moreover, removal of the central glutamine residue leads to a two-orders of magnitude increase of the lifetime of the initially formed spin correlated radical pair, thus corroborating the importance of proton coupled electron transfer (PCET) in the BLUF photocycle (Fudim et al., 2015).

In addition to the resting state photodynamics, also the excited state dynamics and potential photochemical reactions of the signaling state provide information on the changed environment of the chromophore. BLUF domains form the same neutral tyrosyl/flavin radical from the red-shifted state, however with different reaction rates (Toh et al., 2008; Mathes et al., 2012b). In SyPixD the formation is significantly faster and takes place in a seemingly concerted manner. This indicates that the hydrogen bond network in the signaling state of SyPixD is preconfigured for efficient proton transfer to the flavin. Moreover, the initial excited state decay is less heterogeneous, thus indicating a more defined and tighter coordination of flavin and tyrosine in the light-adapted state (Shibata et al., 2009; Mathes et al., 2012b). Similar experiments have been carried out for PapB but tell a slightly different story (Fujisawa et al., 2014). Here, formation of the neutral radical intermediate appears equally fast for light- and dark-adapted states and suggests that the hydrogen bond rearrangement is already accomplished before the formation of the neutral radical. These results suggest that already the formation of a Tyr/FAD charge transfer state may be sufficient to drive the H-bond rearrangement in the flavin binding pocket.

So far, these insights into the dynamics of the photoreaction only provided indirect information on the structural changes involving Gln63 and Trp104. Implications on the Trp orientation have been found for AppA which shows a significant competing, yet signaling incapable ET reaction from Trp104 that leads to a decreased signaling state quantum yield (Laan et al., 2006; Gauden et al., 2007). In SyPixD no competing ET from the corresponding tryptophan has been observed unless the central glutamine is removed (Fudim et al., 2015). For AppA these results suggest that Trp104 is in a proximal position (Trp-in) to the flavin, thus allowing efficient ET. For SyPixD the situation is ambiguos since both Trp conformations (in/out) observed in the crystal structure, are more distant to the flavin than Trp104 in AppA. Moreover, ET from Tyr in SyPixD is significantly faster than in AppA and most likely outperforming Trp also in the Trp-in conformation.

The orientation of the glutamine residue is even more difficult to address based on the currently available spectroscopic data. Even though infrared spectroscopy is in principle capable of detecting the vibrational signature of the glutamine side chain, the strong overlap with the flavin signatures so far prevented any conclusive assignment (Stelling et al., 2007; Bonetti et al., 2008). In the discussion of an early time resolved spectroscopy study on AppA the idea of a tautomerized glutamine has been put forward (Stelling et al., 2007) as an alternative to the already existing rotation mechanism (Gauden et al., 2006) and has been picked up subsequently by computational approaches (Domratcheva et al., 2008; Sadeghian et al., 2008, 2010; Khrenova et al., 2013; Udvarhelyi and Domratcheva, 2013). While tautomeric intermediates are conceivable for the ultrafast reactions, especially in the light-adapted state (Mathes et al., 2012b), their relevance for the long living signaling state remains dubious due to the 10–15 kcal/mol relative instability over the amidic form. Any stabilizing effect of the protein environment for such a species has yet to be found and is an interesting matter for future computational studies.

### Spectrally silent reactions

Although ultrafast spectroscopy showed that the prerequisites for the red-shifted state are already met in less than a nanosecond after excitation, further structural processes that are spectrally silent take place on longer timescale. These changes are likely important for maintaining the signaling relevant conformation for ~10 orders of magnitude longer than its formation. Unfortunately, very limited information is available on any relevant structural changes. In a time resolved FTIR study on the dark recovery of AppA a protonation change of an aspartate residue, most likely the conserved Asp82, was observed on the μs time scale (Majerus et al., 2007) and shows clearly that also peripheral structural changes other than those at the β5 sheet contribute to the stabilization of the signaling state (Figure 1D). Larger scale conformational and oligomeric changes, that have so far been often too challenging to be covered by computational approaches, were observed by transient grating (TG) spectroscopy. Using this technique dimerization or dissociation events were observed (Hazra et al., 2006, 2008; Nakasone et al., 2007, 2010; Tanaka et al., 2009, 2011a,b, 2012; Toyooka et al., 2011) that do not necessarily coincide temporally with the formation or decay of the red shifted signaling state but are dependent on the presence of the conserved methionine residue (Tanaka et al., 2011b).

## Computational studies on BLUF

Here, we summarize the computational approaches, which have addressed these open questions of BLUF domains. The topics covered by those articles are numerous: The Trp conformation is a leading theme for most theoretical studies, but many have ventured further, proposing photocycles (Sadeghian et al., 2008, 2010; Khrenova et al., 2010), structures of intermediates (Domratcheva et al., 2008; Khrenova et al., 2011b), conical intersections (Udvarhelyi and Domratcheva, 2011), and even signal transduction pathways (Khrenova et al., 2011a). These accomplishments have to be acknowledged in the light of the several different time scales on which BLUF events are occurring. The initial excitation process takes only femtoseconds (Frank Condon region), and thus needs treatment completely different from the conformational change of the domain as a whole, which requires up to several hundreds of picoseconds (see above). One has to find the appropriate trade-off between accuracy and computational cost for each of these time scales anew. The fact that we are dealing in several cases with excited state properties is another issue which should be kept in mind, as those properties are computationally much more expensive than ground state properties.

### Computational investigation of the bluf structure

**Figure 2:**
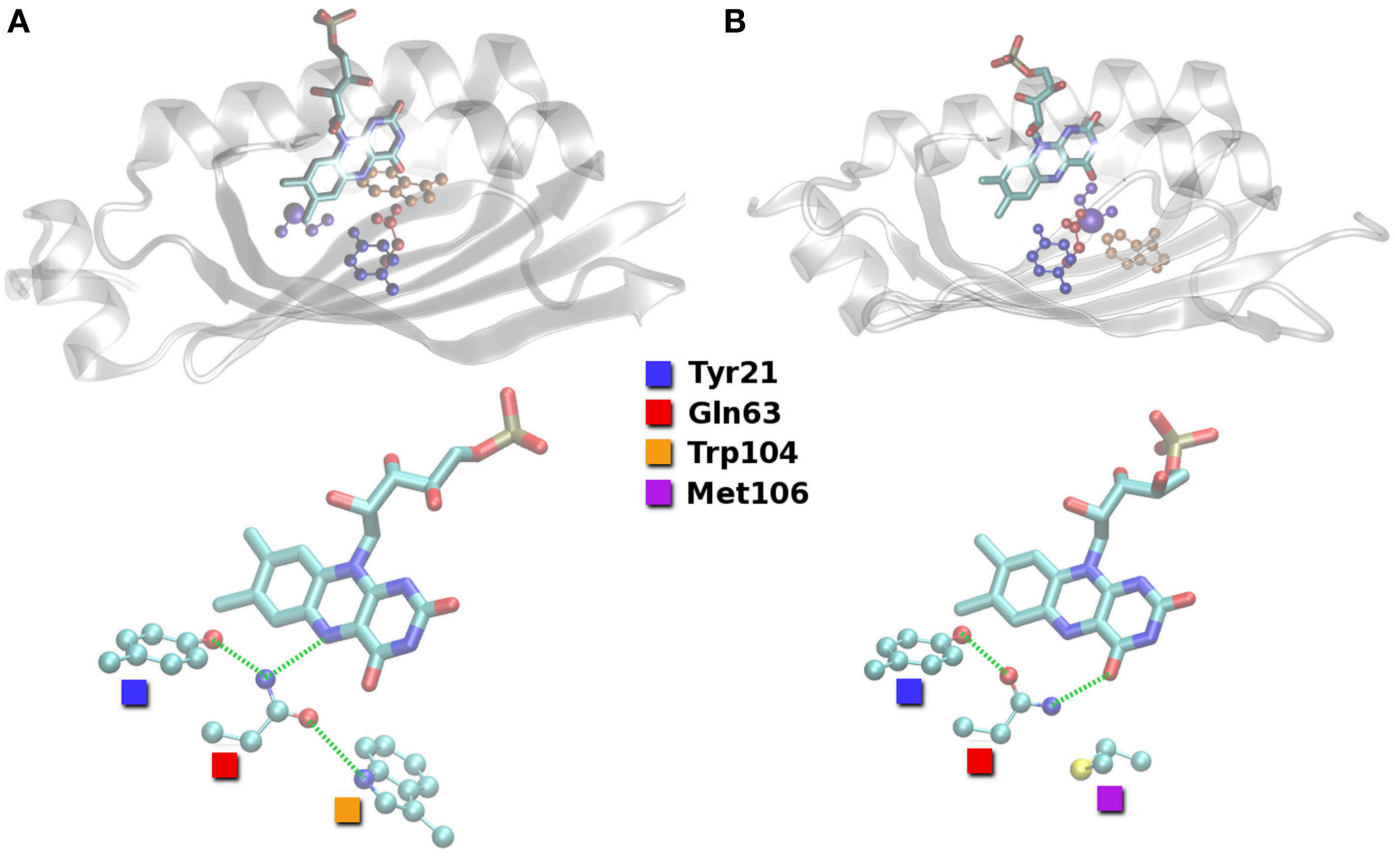
**Hydrogen bond arrangements close to the FMN compound as found in the putative resting state crystal structures of BLUF domains. (A)** Structure of Anderson et al. (2005; PDB ID 1YRX). **(B)** Structure of Jung et al. (2006; PDB ID 2IYG). Hydrogen bond interactions indicated with green dashed lines.

In the past 10 years, several crystallographic, NMR- and vibrational spectroscopic studies have been reported, which aimed at elucidating the molecular details of resting and/or signaling state structure of BLUF domains (see above). However, the assignment to actual orientations of individual amino acids remains contradictory (Figure 2). At least three different (semi-)conserved and functionally relevant amino acids have undefined positions/conformations: Gln63, which is required for the photocycle; and the Trp104/Met106 pair, which is found at different positions in the reported structures (Anderson et al., 2005; Jung et al., 2006). Other amino acids, like Ser41 (Götze and Saalfrank, 2009) and Asn45 (Sadeghian et al., 2008) have been proposed to play a role as well, but those ideas and the associated mechanisms are not as established (and probably not as important) as the basic Gln63/Trp104/Met106 (QWM) triad outlined above. It should be noted that while there are no functional studies on Asn45 available, Ser41 in contrast has been demonstrated to affect ground state spectroscopic properties and dark activity of a BLUF regulated enzyme (Stierl et al., 2014). Interestingly, the quantum yield of signaling state formation is not affected (Bonetti et al., 2009).

The different orientations of the QWM triad are defined by the hydrogen bonding between the Trp104/Met106 pair and Gln63. It is easy to see from Figure 2 that Gln63 is able to accept a hydrogen bond from Trp104 close to FMN (Trp-in). On the other hand, Gln63 is probably a hydrogen bond donor to Met106 inside the binding pocket (Trp-out). It is thus obvious that the region around Trp104/Met106 is flexible to some extent and relevant for signal transduction.

Although a variety of different computational studies have been performed, the comparability between these studies is unfortunately limited due to several reasons: First of all, the variation of BLUF amino acid sequences should be considered. This can be of minor concern, considering exchanges in the terminal or surface areas of the protein, but may also directly influence the comparability of the QM models (like His44 exchanged by arginine in, e.g., BlrB; Zirak et al., 2006, 2007). Hardly comparable to the other published BLUF domain models are studies based on BlrP BLUF (Tyagi et al., 2008; Barends et al., 2009; Wu and Gardner, 2009), since BlrP features a threonine residue instead of Trp104. In general, comparisons between systems with significant sequence deviations should be avoided, both theoretically and experimentally.

A related issue is the variance in the construct length/model size of the BLUF domain. The N-terminal ends are generally neglected, as they are very flexible, and hardly resolved in NMR or X-ray structures. Moreover, a recent experimental work reported the BLUF N-terminus to be of minor importance (Unno et al., 2012). The C-terminus, however, may differ with regards to the presence of the capping α-helices, which follow the β-sheet motif in the BLUF sequence. Those helices are found to be the linker element to the effector domain when applicable (Barends et al., 2009; Khrenova et al., 2011a; Winkler et al., 2013). If present in the BLUF domain crystal, those helices effectively shield the (usually more hydrophobic) β-sheet from the solvent. It should also be noted that the capping helices are differently oriented relative to the β-sheet depending on the BLUF domain at hand (Kita et al., 2005; Yuan et al., 2006; Barends et al., 2009; Wu and Gardner, 2009; Ren et al., 2012). Consequently, molecular dynamics simulations based on structures with those helices included can be expected to perform differently from those simulations which do not contain the C-terminal helices. Structurally resolved C-terminal helices are found in the crystal structures of TePixD (PDB ID 1X0P), SyPixD (PDB ID 2HFN), and all of the full length BlrP and AppA protein structures (Barends et al., 2009; Winkler et al., 2013). Some computational studies ignore the problem of varying sequence length by simply using the crystal structures as they are found in the PDB database (Domratcheva et al., 2008; Sadeghian et al., 2008; Hsiao et al., 2012); others cut the chains to analogous lengths to obtain constructs of equal size (Götze et al., 2012; Meier et al., 2012). As some BLUF domains occur in dimers or higher oligomers in solution (Hazra et al., 2006; Nakasone et al., 2007; Tanaka et al., 2009), another approach is to use dimeric models for those structures where the C-terminal helices are not reported and a dimerization interface is derived from the X-ray crystal structure (Rieff et al., 2011).

A possibly crucial, yet underappreciated issue is the amino acid protonation state. Crystal structures do not resolve hydrogen atoms, and NMR studies have been found to be contradictory in some regards (as discussed in Rieff et al., 2011). The resulting problem is two-fold: First, the signaling state Gln63 residue might be present in a tautomerized form (Stelling et al., 2007), which will be addressed below. Second, the histidine protonation state is undefined. Depending on the individual BLUF sequence, the reported structures contain up to five His residues (AppA: 5; BlrP: 4; BlrB, SyPixD, TePixD: 3). While the protonation state of buried residues may possibly be derived from the immediate environment, surface located His residues might either be fully protonated, depending on the pH, or vary between δ- or ε-protonated, neutral tautomers. Most modeling approaches in the past did not account for the possible variance in His protonation and assumed an arbitrary, static His protonation, with few exceptions (Rieff et al., 2011; Götze et al., 2012). The different His protonation schemes throughout the literature prevent for a direct 1:1 comparison of the individual results.

Finally, we would like to shift the focus away from the static atomic modeling problem to a dynamic view of the thermal BLUF movement. In the light of the MD studies conducted, which analyzed the hydrogen bonding probabilities inside the BLUF FMN binding pocket (Obanayama et al., 2008; Götze et al., 2012; Meier et al., 2012), the “on-off” picture of the QWM triad becomes more blurry, especially for the Trp-in conformations. In Götze et al. (2012), it was proposed that already the choice of the initial velocities can significantly influence the MD hydrogen bonding behavior of Gln63. While the results between the available MD studies are often not in line with each other (Obanayama et al., 2008; Meier et al., 2012), the diversity of results throughout the literature clearly indicates that a static, “zero Kelvin, QM only” view of the BLUF domain could be inappropriate for such a dynamic system. At least including temperature effects on a minimal level might be required in order to obtain meaningful results. For QM cluster models, a valid alternative would be to compare QM studies preferably to experimental low-temperature studies (see Fukushima et al., 2007, 2008).

### Computational approaches

From the previous section, it becomes clear that molecular models of BLUF domains are always associated with various assumptions. Despite these shortcomings, computational groups have made substantial progress regarding two core questions:

- *Photodynamics*: How are the different kinetic components of the photocycle related to physical processes in the protein matrix and the chromophore?
- *Structure*: How are the different experimental characteristics combined to form a resting or signaling state conformation?

Since we focus below on the structural flexibility and spectroscopic properties of BLUF domains, there are some computational studies that we cannot take into account for our analysis. Those are the studies that do not provide explicit computations of hydrogen bonding probabilities and UV/vis or IR spectra (Ishikita, 2008; Nunthaboot et al., 2009, 2010).

The photodynamics research branch employs the Trp-out BLUF resting state conformation presented by Jung et al. (2005, 2006; PDB ID 2IYG). All models assume the Trp-in conformation as the signaling state. This assignment is supported by the full length structure of AppA, which displays a Trp-out conformation in the putative resting state (Winkler et al., 2013). However, there is a catch: Experimental evidence indicates a stronger hydrogen bond to FMN(O4) from the protein matrix in the signaling state, due to the drop of the FMN(C4)-FMN(O4) force constant upon signaling state formation (Masuda et al., 2005b). A stronger hydrogen bond cannot be explained by a chemically unaltered QWM triad, as Gln63 is required to present its oxygen atom toward Trp104 in a Trp-in-containing signaling state. Hence, the hydrogen bonding of Gln63(NH2) to FMN(O4) would be impossible.

To overcome this contradiction, Gln63 can be assumed to be a tautomer in the Trp-in signaling state (Stelling et al., 2007). This was first suggested by an experimental investigation (Stelling et al., 2007) with the caveat that the tautomer is expected to be about 10–15 kcal/mol less stable than the amide form. Imidic Gln63 is able to form (i) a donor hydrogen bond to FMN(O4) and (ii) an acceptor hydrogen bond to Trp104 at the same time. This would allow Gln63 in a Trp-in conformation to be a (possibly stronger) hydrogen bond donor to FMN(O4) than in the Trp-out resting state, consistent with the experiment. Yet, as tautomerized Gln63 is a possible hydrogen bond donor and acceptor on *both* the N and the O atoms, this feature is independent of the Gln63 rotational orientation. This rotation, which might or might not occur upon signaling state formation, is the central difference between the two models based on imidic Gln63 and a Trp-out resting state. The models presented by Domratcheva et al. include a Gln63 rotation during signaling state formation (Domratcheva et al., 2008; Khrenova et al., 2010, 2011b; Udvarhelyi and Domratcheva, 2011), while Sadeghian et al. assume a more rigid orientation of Gln63 at all times (Sadeghian et al., 2008, 2010). How this translates to differences in the proposed photocycles will be the topic of an individual section further below.

### Structural models

As a consequence from the QWM triad uncertainties, several groups tried to reproduce the experimental findings outlined above using calculated spectra and molecular dynamics. Since the observed shifts in UV/vis and IR signals are from a computational point of view rather small (about 0.1 eV or 20 cm^−1^, respectively), only qualitative answers can be expected from these approaches. Concerning pure MD studies, which provide multiple ns trajectories of BLUF domains in different conformations, the reports by Khrenova and a study by Meier (Khrenova et al., 2011a; Meier and van Gunsteren, 2013) would fit into this group. However, there is no analysis of the hydrogen bonding patterns close to the flavin chromophore, as Khrenova et al. only feature hydrogen bonding patterns focused on the interaction of the BlrP BLUF domain and its target protein effector domain.

Unfortunately, the results of the remaining articles are hardly comparable. All other MD studies contain, possibly among others, the BLUF domains of PDB entries 1YRX and 2IYG (AppA Trp-in and Trp-out), but the reported hydrogen bonding patterns are very much different (see Table 1). The studies feature different force fields, equilibration protocols, run parameters, and histidine protonation states; it is therefore impossible to provide a convincing explanation for the strong disagreement of the data.

**Table 1:** **AppA BLUF hydrogen bonding patterns as displayed in Obanayama et al. (2008), Götze et al. (2012), and Meier et al. (2012)**.

**Reference and year**	**Hydrogen bonding ratio/%**							
	**Tyr21(OH)–Gln63(O)**		**Gln63(NH)–FMN(O4)**		**Gln63(NH)–FMN(N5)**			
	**Trp-in**	**Trp-out**	**Trp-in**	**Trp-out**	**Trp-in**	**Trp-out**		
Obanayama et al., 2008	80.0	62.7	33.8	10.0	16.1	28.0		
Meier et al., 2012^a^	0.0/0.0	4.5/92.8	81.4/45.2	50.4/76.9	44.7/56.4	53.1/17.8		
Götze et al., 2012^b^	25.0 ± 31.3	85.6 ± 6.2	5.0 ± 5.3	0.7 ± 0.6	1.2 ± 1.1	2.2 ± 1.2		
	**Asn45(NH)–FMN(O4)**		**FMN(N3H)–Asn45(O)**		**Gln63(NH)–Tyr21(O)**		**Trp104(NH)–Gln63(O)**	
	**Trp-in**	**Trp-out**	**Trp-in**	**Trp-out**	**Trp-in**	**Trp-out**	**Trp-in**	**Trp-out**
Meier et al., 2012^a^	84.7/94.0	2.0/15.8	86.6/86.8	77.5/94.1	93.0/99.2	0.1/0.0	54.0/90.1	0.0/0.0
Götze et al., 2012^b^	21.7 ± 6.7	16.1 ± 7.7	27.4 ± 7.7	34.9 ± 11.8	3.2 ± 7.9	0.0 ± 0.0	12.4 ± 20.2	0.0 ± 0.0

Hydrogen bond criteria, model construction and computational methodology may differ.

a Two force fields (Gromos45A4/Gromos53A6) were used.

b Standard deviations from eight trajectories per model.

A second group of articles, partially overlapping with the first, is focusing on the generation of UV/vis and IR spectra from (post-processed) MD snapshots, X-ray or NMR data (Obanayama et al., 2008; Götze and Saalfrank, 2009; Rieff et al., 2011; Götze et al., 2012; Hsiao et al., 2012). Obanayama et al. (2008) belong to this group, although the presented spectra are based on a single structure, which is statistically problematic. Additionally, another study considers TD-DFT QM cluster spectra, based on NMR geometries, on manually created conformations and also MD snapshots (Götze and Saalfrank, 2009). A unique approach in structure generation is taken elsewhere, for which dynamic DFT/MM calculations are employed, obtaining IR spectra from protein dynamics (Rieff et al., 2011). Regarding the MD methodology, Rieff et al. (2011) is very close to the first group of articles; yet the authors do not present any hydrogen bonding patterns in their report. They do, however, find that the quality of the published NMR and X-ray structures is very heterogeneous, and conclude that the structures 1YRX and 2IYG represent the more reliable ones. Another article presents DFT/MRCI (Grimme and Waletzke, 1999) excitations on QM refined X-ray and artificial structures (Hsiao et al., 2012). Additionally to the trajectories discussed above for the data in Table 1, Götze et al. (2012) contains TD-DFT spectra on the basis of MD snapshots. A more recent member of this group contains data on both native and isomerized Gln63 (see next section) (Udvarhelyi and Domratcheva, 2013). Finally, the latest article trying to elucidate the structure of the BLUF resting state, features a simplified variant for reproducing exclusively the shifts of the UV/vis spectra upon changing the BLUF domain structure, e.g., by mutation (Collette et al., 2014).

Table 2 lists the calculated UV/vis and IR signals, for which a direct comparison between Trp-in and Trp-out is made within the same article. Experimental data are given for comparison, related to resting and signaling state. In Rieff et al. (2011), introduction of a polarizable force field essentially inverts the corresponding results for the IR spectrum, and the TD-DFT spectra of Götze et al. (2012) are non-conclusive. The results shown in (Götze and Saalfrank, 2009; Hsiao et al., 2012; Udvarhelyi and Domratcheva, 2013) agree that the Trp-in QWM triad displays a blue-shifted absorption spectrum compared to the Trp-out conformation. This notion is supported by Obanayama et al. (2008), although the data presented are based on a single structure only, as already indicated above. Note that the authors of Udvarhelyi and Domratcheva (2013) conclude that an imidic Gln63 residue is responsible for the signaling state (see below), and do not follow the assignment that we suggest here by the arrangement of Table 2.

**Table 2:** **AppA BLUF UV/vis and IR signals, as calculated throughout the literature from MD, NMR, or crystal structures**.

**Reference and Year**	**UV/vis maxima**			
	First FMN π → π^*^		Second FMN π → π^*^	
	**Trp-in**	**Trp-out**	**Trp-in**	**Trp-out**
Obanayama et al., 2008	426 nm	435 nm	n/a	n/a
Götze and Saalfrank, 2009	448 nm	459 nm	339 nm	350 nm
Hsiao et al., 2012^a^	523 nm	575 nm	380 nm	405 nm
	441 nm	460 nm	355 nm	370 nm
Götze et al., 2012	438 nm	437 nm	347 nm	347 nm
Udvarhelyi and Domratcheva, 2013^b^	435 nm	(448 nm)	n/a	n/a
	443 nm	(444 nm)		
	432 nm	(437 nm)		
	**Resting**	**Signaling**	**Resting**	**Signaling**
Exp. Kraft et al., 2003	446 nm	458 nm	373 nm	378 nm
	**IR FMN(C4 = O4) stretch**			
	**Trp-in**	**Trp-out**		
Obanayama et al., 2008	1700 cm^−1^	1689 cm^−1^		
Rieff et al., 2011^c^	1750 cm^−1^	1739 cm^−1^		
	1724 cm^−1^	1733 cm^−1^		
	**Resting**	**Signaling**		
Exp. Masuda et al., 2005b	1709 cm^−1^	1695 cm^−1^		

Models with imidic Gln63 not included.

a Hsiao et al. (2012) reports several other structures in qualitative agreement with the presented structures. MD structure reported here (second line) corresponds to QM refined X-ray structure with weighting factor w_xref_ = 1 (see reference, for details).

b Three different optimization restraint settings reported. No actual Trp-out structures, only rotated Gln63. In this study Trp-in is not considered to be the resting state.

c Reported values for static (upper line) and polarizable environment (lower line). Rieff et al. (2011) also reports rescaled values, about 20 cm^−1^ lower.

The recent article by Collette et al. employs a different approach that does not lead to explicit values for the absorption wavelengths that could be compared to the other studies (Collette et al., 2014). Instead, they focus on the computation of the shifts that occur upon mutation, or the introduction of imidic Gln63 (iGln63) in different conformations. Their results unanimously prefer the Trp-in conformation to be the signaling state, due to the qualitative and quantitative comparability of the computed shifts to the experimental measurements. The authors have, however, not performed a thorough reoptimization of their structures before computing the shifts after insertion of the mutant/modified amino acid, thereby neglecting any influence that an exchange has on neighboring residues (or even further away from the point of exchange). As such, the data is drawn from a basis of BLUF domains in which the mutations have a strongly localized effect, and no effect on the overall protein or FMN conformation, which is not very likely.

Apart from this latest article in the field, it appears that the structural studies favor Trp-in to be the resting state; yet the hydrogen bonding patterns presented do not fully agree with each other and the calculated IR data are sparse. The computed UV/vis spectra are, however, consistent. In the following section we will discuss the influence of imidic Gln63.

### Photocycle models

Assuming Gln63 to form an imide (iGln63) upon FMN excitation provides an alternative to form hydrogen bonds to FMN(O4) in a Trp-in signaling state (see above). As no standard force field parameters for iGln63 are available, reported models of BLUF domains with iGln63 mostly rely on QM cluster or QM/MM approaches with iGln63 in the QM part (Domratcheva et al., 2008; Sadeghian et al., 2008, 2010; Khrenova et al., 2010, 2011b; Udvarhelyi and Domratcheva, 2011). Merz et al. (2011) technically belongs to that group as well, but investigates the effect of a FMN replacement by roseoflavin, and will therefore not be discussed further. Most photocycle studies discuss the Trp-in/Trp-out problem to some extent, but all presented models assume Trp-out (PDB ID 2IYG) to be the resting state, and Trp-in is disregarded as a resting state candidate by initial comparison of structural and experimental data. Consequently, the focus of iGln63 models is not the Trp-in/Trp-out dilemma, but the photocycle of BLUF domains. A notable exception is reference (Udvarhelyi and Domratcheva, 2013), where the 1YRX Trp-in structure was used as the basis for a study involving iGln63.

Just as the structural articles of the last section, the reported excitation energies produce the required ~10 nm shift in the UV/vis spectrum, despite the inverted conformation/state assignment. Furthermore, the calculated IR spectra display (for the most cases) the experimentally shown shift to lower energies in the FMN(C4 = O4) stretching vibration. On the downside, the second π → π^*^ excitation is rarely reported or discussed, and the computed UV/vis signals of the iGln63 models are overall shifted to higher energies. Extremely puzzling is that there is little overlap between Table 2 and Table 3 in the case of the Trp-out conformation although the chemical composition of the Trp-out cases is identical. Yet, the Trp-out values in Table 3 are uniformly blue-shifted compared to the ones of Table 2. A probable reason is that QM methods differ significantly; in the articles summarized Table 2, mostly DFT based methods have been employed, while iGln63 models feature mostly CC2 or CASSCF-related calculations. As those are not the only differences, we again end up with the problem of comparability between the works of different groups, which already hampered our analysis before.

**Table 3:** **BLUF UV/vis and IR signals, as calculated from imidic Gln63 models throughout the literature**.

**Reference and Year**	**UV/vis maxima**			
	First FMN π → π^*^		Second FMN π → π^*^	
	**Trp-out**	**Trp-in**	**Trp-out**	**Trp-in**
Domratcheva et al., 2008	447 nm	454 nm	361 nm	356 nm
Sadeghian et al., 2008^a^^,^^b^	377 nm	390 nm	n/a	n/a
	414 nm	425 nm		
Khrenova et al., 2010	425–429 nm	441 nm	n/a	n/a
Khrenova et al., 2011b	424 nm	442 nm	n/a	n/a
Udvarhelyi and Domratcheva, 2013^c^	(444 nm)	461 nm	n/a	n/a
	(437 nm)	451 nm		
	**Resting**	**Signaling**	**Resting**	**Signaling**
Exp. AppA Kraft et al., 2003	446 nm	458 nm	373 nm	378 nm
Exp. BlrB Zirak et al., 2006	445 nm	457 nm	374 nm	379 nm
	**IR FMN(C4 = O4) stretch**			
	**Trp-out**	**Trp-in**		
Domratcheva et al., 2008	1742 cm^−1^	1748 cm^−1^		
Sadeghian et al., 2008^a^	1798 cm^−1^	1791 cm^−1^		
Khrenova et al., 2010	1700 cm^−1^	1675 cm^−1^		
	**Resting**	**Signaling**		
Exp. AppA Masuda et al., 2005b	1709 cm^−1^	1695 cm^−1^		
Exp. BlrB	n/a	n/a		

AppA BLUF if not noted otherwise. Note that Trp-in and Trp-out columns have been switched compared to Table 2 as the consensus assignment of the resting and signaling state is different.

a BlrB BLUF (PDB ID 2BYC).

b Reported values for BHLYP (upper line) and CC2 (lower line).

c Two different optimization restraint settings for the iGln63 configurations reported. Trp-out values taken from Table 2; no actual Trp-out structures, only rotated Gln63.

The greatest strength of the iGln63 models is that they provide possible photocycle mechanisms that relate to the experimentally observed PCET mechanism between Tyr21 and the flavin (see above). Computationally, a drop in energy is reported when following a Tyr21-Gln63 proton transfer in the lowest charge transfer state (CT) (Sadeghian et al., 2008). This CT may be populated by the bright π → π^*^ transition locally excited state (LE) of FMN. The proposed pathway involves the excitation of the LE state, crossing to the CT state and subsequent proton transfer from Tyr21(OH) to Gln63(O). The LE-CT conical intersection (CIn) close to the Franck-Condon (FC) region was structurally identified, having reduced Tyr21(OH)-Gln63(O) and Gln63(NH_2_)-FMN(N5) hydrogen bond distances compared to the FC geometry (Udvarhelyi and Domratcheva, 2011). The initial proton transfer is followed by a second transfer from Gln63(NH_2_) to FMN(N5), crossing back to a Tyr21^•^-iGln63-FMNH^•^ biradical ground state along the reaction coordinate. Here, the corresponding CIn was found to be structurally elusive.

Up to this point, the articles following the idea of an iGln63 are in agreement, afterwards the paths deviate: Sadeghian et al discard the possibility of Gln63 rotation, and they assume a Tyr21(O)-Gln63(O) hydrogen bond to be formed at all times. Assuming a *trans*-*cis*-isomerization of the iGln63(NH^−^) group, the moiety is allowed to become a hydrogen bonding partner for FMN(O4). They find the *cis*-conformer of iGln63 to be more stable in a Trp-in conformation, and consequently assume conformational switching of Met106 and Trp104 upon formation of *cis*-iGln (Tanaka et al., 2009). They also investigated the possible conformations of a water molecule close to Tyr21, which was required for the Tyr21 conformation to remain stable (a possibly different reason is described in Rieff et al., 2011). Subsequently, a model for the events in the light-adapted state was proposed, explaining how the light-adapted conformation relaxes directly back to the ground state (Sadeghian et al., 2010). Two channels supposedly exist, based on either partial or complete, possibly concerted proton transfer involving Tyr21(OH), iGln63(OH^+^), and FMN(N5). Both channels essentially recombine quickly to the ground state, explaining the photo-irreversibilty of the signaling state.

A different route was proposed by Domratcheva and coworkers. They proposed iGln63 rotation before or during the generation of the Trp-in conformation. This notion is based on structural arguments, supported by MD simulations and the corresponding QM/MM energies (Khrenova et al., 2011b). As already above, iGln63 essentially allows to form a hydrogen bond to FMN(O4) in both rotamers; therefore it might be very well possible that both mechanisms are taking place. Yet, it should be noted that the missing Tyr(OH)-Gln63(O) hydrogen bond in the signaling state may be in contrast to a recent experimental article (Iwata et al., 2011), and must possibly be replaced by a strong Tyr(OH)-iGln63(N) interaction. Recently, Domratcheva and coworkers have compared their mechanisms to the pathways proposed by the Schütz group; they concluded that a pathway including iGln63 rotation is favored (Khrenova et al., 2013).

So far, only one article provides insight in the actual Trp/Met exchange process on the basis of molecular dynamics and structural conversion form Trp-in to Trp-out and vice versa on the level of force fields (Meier and van Gunsteren, 2013). However, a viable explanation how the global BLUF conformation reacts on the relatively small changes close to the FMN chromophore is still missing (cf. Peter et al., 2012 for the LOV domain case). As this is an essential part of the photocycle and is expected to explain the relatively high stability of the hydrogen bond switched state, the questions regarding the BLUF photocycle are only partially answered. Furthermore, it would be required to test whether a reasonable photocycle can be constructed with the Trp-in conformation as well. So far two possible photocycles have been proposed for the Trp-out conformation, but especially due to the lack of calculations for a potential Trp-in based photocycle predictions that can be confirmed experimentally are not available from the computational studies.

## A photocycle for the trp-in resting state

In this section, we will present the results of basic density functional theory (DFT) calculations, serving as a preliminary framework for a potential photocycle for a Trp-in resting state. The calculations are based on small clusters taken from the 1YRX and 2IYG (AppA Trp-in/Trp-out) crystal structures, including lumiflavin, Tyr21, Ser41, Gln63, and Trp104/Met106. A detailed setup of the calculations can be found in the Supporting Information.

First, we tested the influence of Grimme's dispersion correction (Grimme et al., 2010) on the molecular interaction energies. This dispersion correction was recently introduced and refined over the years to remove the shortcomings of density functional theory when it comes to the interaction of weakly bound molecular clusters. The reason behind this is that DFT tends to underestimate the actual interaction between compounds that are not bound via covalent bonds or static Coulomb interactions (ions, dipoles). The presented values were calculated from a Counterpoise corrected optimization (Boys and Bernardi, 1970), including geometry restraints to keep the protein structure roughly resembled in our cluster models. Strikingly, the dispersion correction has a strong influence on the stability of Tyr-FMN and Ser-FMN interactions. In both cases, the interaction is quantitatively affected by up to 5 kcal/mol (see Table 4).

**Table 4:** **Interaction energies (in kcal/mol) of Tyr21 and Ser41 with the rest of the investigated BLUF model**.

**Geometry**	**E**_*****I*****_**(Tyr21)**			**E**_*****I*****_**(Ser41)**		
	**w/D3BJ**	**w/o D3BJ**	**ΔE**	**w/D3BJ**	**w/o D3BJ**	**ΔE**
A w/D3BJ	−7.16	−2.68	−4.48	−9.09	−4.26	−4.83
A w/o D3BJ	−7.09	−2.82	−4.27	−8.65	−4.19	−4.46
J w/D3BJ	−15.03	−10.09	−4.94	−5.21	−0.88	−4.33
J w/o D3BJ	−15.10	−10.25	−4.85	−5.36	−1.12	−4.24

A-values are based on the Anderson et al crystal structure (PDB ID 1YRX; Anderson et al., 2005) and J-values on the structure of Jung et al. (2006; PDB ID 2IYG). D3BJ indicates the presence or absence of the dispersion correction in the CAM-B3LYP calculations.

This shows that studies using DFT are limited in obtaining the correct binding energies and conformations for the BLUF domain when dispersion correction is not considered. This is particularly relevant for BLUF due to the large aromatic ring system of FMN and its potential π-interactions perpendicular to the isoalloxazin plane, which have still not found much attention in the literature.

**Figure 3:**
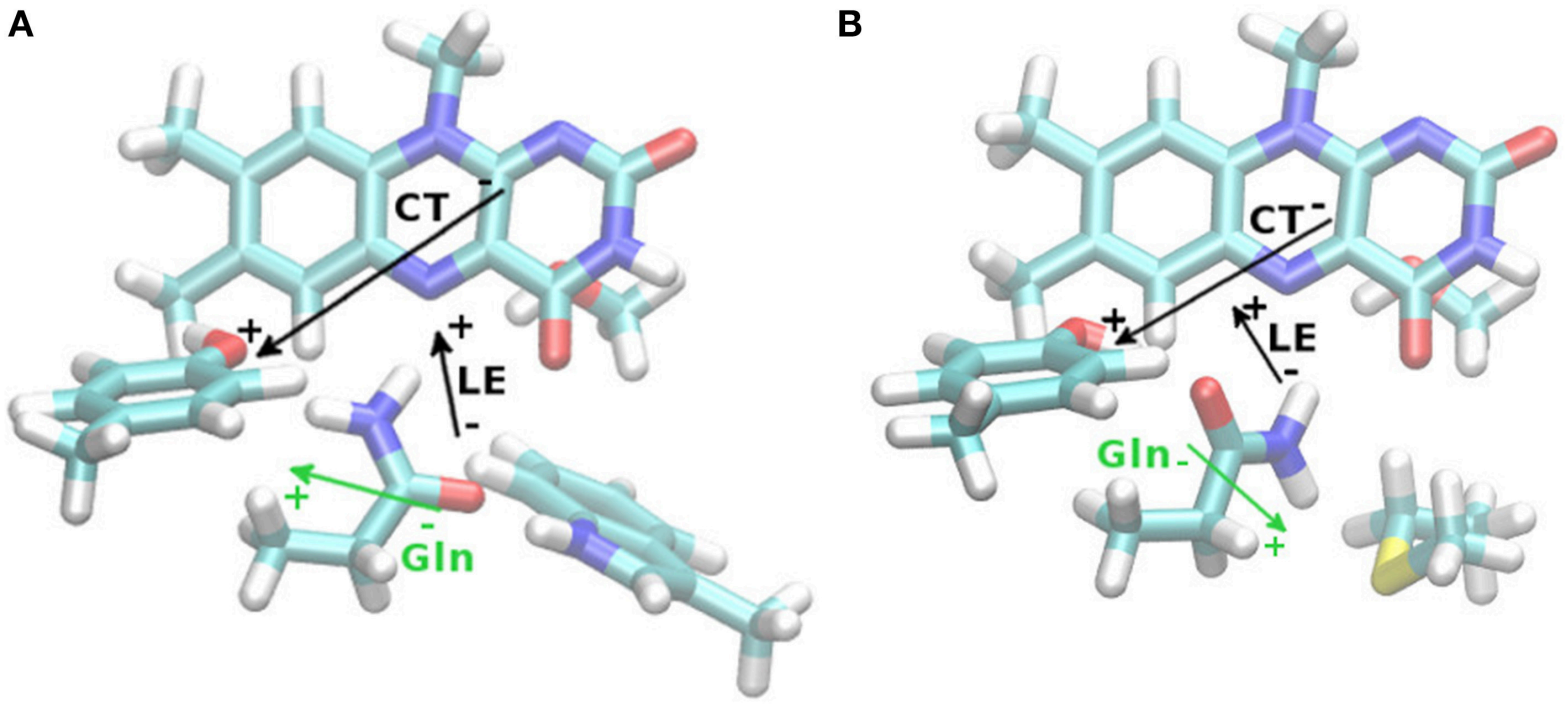
**TD-DFT difference dipole vectors (|μ_t_–μ_0_|, black arrows) based upon the Anderson (A) and Jung (B) CAM-B3LYP/6-31G(d,p) structures with D3BJ correction, for the lowest local excited state (LE) or the Tyr-FMN charge transfer (CT) state**. Lengths of vectors are arbitrary, for actual size refer to Table 5. Gln63 permanent ground state dipole moment shown as well (green arrows).

Second, we conducted an analysis of the change in dipole moment upon excitation using a long-range corrected density functional, CAM-B3LYP (Yanai et al., 2004). In the excited system, the change in dipole moment is between 2 and 33 Debye for the investigated states (local excited or charge transfer state, respectively, see Table 5). For comparison to Table 2, the TD-CAM-B3LYP vertical excitation energies for the bright state of the Anderson/Jung structures are 371/374 nm (regardless of dispersion correction), resulting in a small red shift of 0.03 eV. Given the nature and the physical meaning of vertical excitation energies (namely an upper limit to the range in which the photophysics take place, Götze and Thiel, 2013; Karasulu et al., 2014), the apparent “overestimation” resulting from the CAM-B3LYP calculations is indeed more realistic than the reproduction of a measured absorption maximum. While it is experimentally established that the electron transfer from Tyr21 to FMN leads to the formation of the red-shifted species, it has so far not been considered that the resulting change in local dipole moment might already strongly perturb the hydrogen bond network around the flavin. The MD simulations agree that the Gln63 conformation in the BLUF domain is very flexible (Obanayama et al., 2008; Götze et al., 2012; Meier et al., 2012). As such, transferring an electron to FMN and thus establishing a strong polarized electric field is likely to change the conformational distribution of Gln63. More precisely, the very initial effect of the electron relocation form Tyr21 to the polar part of FMN is the formation of a reversed electric field in the vicinity of the Gln63 side chain (Figure 3). Since the Gln63 side chain is a permanent dipole itself, a reaction of the Gln63 dynamics to the changed electric field can be expected, such as the proposed Gln63 flip.

**Table 5:** **Changes in dipole moment (in Debye) upon vertical excitation to the lowest bright local excitation (LE) or to the Tyr-FMN charge transfer (CT) state**.

	**A w/D3BJ**		**A w/o D3BJ**		**J w/D3BJ**		**J w/o D3BJ**	
	**|μ_t_|–|μ_0_|**	**|μ_t_–μ_0_|**	**|μ_t_|–|μ_0_|**	**|μ_t_–μ_0_|**	**|μ_t_|–|μ_0_|**	**|μ_t_–μ_0_|**	**|μ_t_|–|μ_0_|**	**|μ_t_–μ_0_|**
LE, Gas phase	0.02	1.70	−0.05	4.18	0.44	1.98	0.39	1.98
LE, CPCM	0.96	2.03	1.15	2.28	1.05	2.38	0.95	2.36
CT, CPCM	28.00	33.15	27.56	33.10	24.20	31.73	24.21	31.85

**Figure 4:**
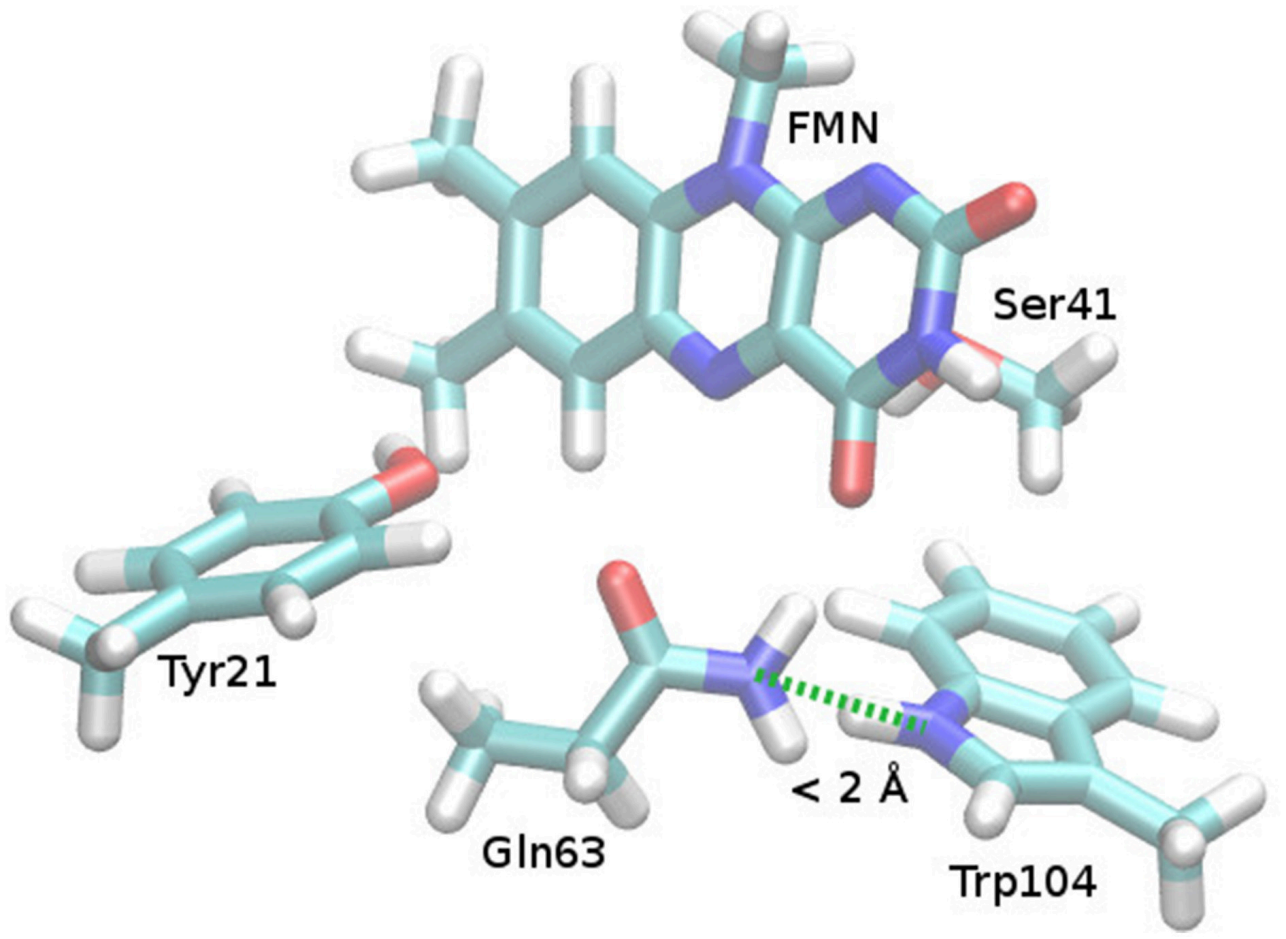
**Repulsive steric interaction between Gln63 and Trp104 after Gln63 rotation**. Cut from the Anderson et al. AppA BLUF structure (PDB ID 1YRX, Anderson et al., 2005), missing hydrogen atoms added and optimized (see Supporting Information). After preparation, the Gln63 orientation was manually adapted to the torsional angles found in the structure of Jung et al. (2006; PDB ID 2IYG), yielding the presented structure.

In case of a flipped Gln63 anchor, there is no reason for the Trp104 to remain in the FMN binding pocket. There is no way for Gln63-NH_2_ and Trp104-NH to interact in a binding fashion and even a slight steric repulsion can be inferred (see Figure 4). This might lead to a small preference for the Trp-out conformation, where Gln63 can form a hydrogen bond to the Met106 sulfur. This mechanism leaves the possibility for a post-rotation proton transfer from Tyr21 to FMN, either via Gln63 or directly, and enables the formation of the red shifted state immediately after the formation of the CT state.

## A perspective for BLUF research

Although numerous experimental studies on BLUF photoactivation and its dynamics are available so far, a clear picture of the molecular processes in BLUF domain remains elusive. The commonly accepted features of BLUF photosensing consist of PCET driving a hydrogen bond rearrangement and that the structural properties of the β5 strand involving the conserved methionine and possibly also the semi-conserved tryptophan (where present) are essential for signal transduction. Orientation and photoinduced dynamics of the conserved glutamine, tryptophan and methionine side chains, however, are still far from understood. Moreover, secondary structural changes are most likely required for the high stability of the hydrogen bond switched state but this aspect has not been addressed sufficiently so far. To solve these fundamental questions in the future, experimental approaches sensitive and selective for individual chemical groups in the protein have to be applied. It also appears obvious that classical site-directed mutagenesis and the use of truncated proteins are very limited in providing a unifying picture of photoactivation and signal transduction of the wild type proteins. A promising approach in this regard is to use group/site-specific isotope labels in combination with time resolved vibrational spectroscopy methods like transient IR absorption, two-dimensional IR spectroscopy or stimulated Raman spectroscopy. The resulting data would furthermore provide a basis for theoreticians to test/benchmark their calculations and to validate potential models.

The differences in the field of theory could be easily amended, but any attempt for consolidation has to take into account that the existing models already cover a wide variety of methods. A neutral point of view comparing Trp and Gln conformations and predicting observables that can be tested experimentally would be ideal. While several computational studies claim that they adopt a neutral point of view, they still present a specific model as their favorite as outcome. Given the usual error ranges of the employed theoretical methods (e.g., DFT methods 0.2–0.5 eV, Silva-Junior et al., 2008), these conclusions appear tentative at best. Further progress therefore can therefore only be made by advanced experimental approaches in combination with qualitative theoretical predictions.

### Conflict of interest statement

The authors declare that the research was conducted in the absence of any commercial or financial relationships that could be construed as a potential conflict of interest.

## Footnotes

^1^Resting/signaling state is the notation which will be used throughout the article, in contrast to the commonly used “dark/light” terminology; we seek to avoid confusion with optically “dark” or “bright” electronic states.
